# Novel Insights into the Inheritance of Gibberella Ear Rot (GER), Deoxynivalenol (DON) Accumulation, and DON Production

**DOI:** 10.3390/toxins14090583

**Published:** 2022-08-24

**Authors:** Akos Mesterhazy, Balázs Szabó, Sándor Szél, Zoltán Nagy, Attila Berényi, Beata Tóth

**Affiliations:** Cereal Research Non-Profit Ltd., H-6726 Szeged, Hungary

**Keywords:** maize, resistance to Gibberella ear rot, heterosis for Gibberella ear rot, inheritance of the resistance to toxin accumulation, DON-producing intensity

## Abstract

Gibberella ear rot (GER) is an important fungal ear pathogen of maize that causes ear rot and toxin contamination. Most previous works have only dealt with the visual symptoms, but not with the toxins of GER. As food and feed safety rankings depend on toxin contamination, including deoxynivalenol (DON), without toxins, nothing can be said about the risks involved in food and feed quality. Therefore, three susceptible, three medium-susceptible, and three medium-resistant mother lines were crossed with three testers with differing degrees of resistance and tested between 2017–2020. Two plot replicates and two fungal strains were used separately. The highest heterosis was found at the GER% with a 13% increase across 27 hybrids, including 7 hybrids showing negative heterosis (a higher hybrid performance above the parental mean), with a variance ranging between 63.5 and −55.4. For DON, the mean heterosis was negative at −35%, and only 10 of the 27 hybrids showed a positive heterosis. The mean heterosis for DON contamination, at 1% GER, was again negative (−19.6%, varying between 85% and 224%). Only 17 hybrids showed heterosis, while that of the other 17 was rated higher than the parental mean. A positive significant correlation was found only for GER% and DON; the other factors were not significant. Seven hybrids were identified with positive (2) or negative (5) heterosis for all traits, while the rest varied. For DON and GER, only 13 provided identical (positive or negative) heteroses. The majority of the hybrids appeared to diverge in the regulation of the three traits. The stability of GER and DON (variance across eight data sets) did not agree—only half of the genotypes responded similarly for the two traits. The genetic background for this trait is unknown, and there was no general agreement between traits. Thus, without toxin analyses, the evaluation of food safety is not possible. The variety in degrees of resistance to toxigenic fungi and resistance to toxin accumulation is an inevitable factor.

## 1. Introduction

Gibberella ear rot (GER) is a severe ear rot disease. It can cause significant ear rot and a decrease in the yield, but high mycotoxin contamination by deoxynivalenol (DON) and zearalenone (ZEA) and their derivatives can destroy the financial value of the whole yield. The latter occurs less frequently; following warm weather before harvest, it is normally not found. In later hybrids, under cool and rainy weather conditions, the zearalenone risk increases, as observed in Hungary in 2010 and 2014. Regarding toxicity, Han et al. [1] summarized the latest information; however, nothing was mentioned about the possible role of plant resistance in reducing ZEA contamination in maize. As DON and GER are closely correlated in most hybrids [2,3,4], we consider that it is worth identifying the role of plant resistance to *F. graminearum* and to ZEA contamination. GER is dangerous in moderately warm and humid seasons. As ZEA requires cool weather for synthesis, during storage, DON-contaminated grains can additionally be severely contaminated by ZEA, even if, at harvest, no sign of its presence could be found.

The causative agent is *Gibberella zeae* (Schwabe) Petch. and its imperfect form, *F. graminearum* Petch. In the infection process, both ascospores and conidia play a role. Previously, it was considered as a necrotrophic pathogen attacking physiologically weakened plants. However, this may not be the case, especially when GER affects maize fields with robust plants and productions above 10 tons/ha. Therefore, severe infection is instead due to susceptibility [2,4], e.g., a lack of resistance. For instance, in wheat, it was considered that the fungus infection starts by exhibiting a biotrophic lifestyle [5,6], and after 3–4 days, it switches to a necrotrophic lifestyle. As *F. graminearum* is highly aggressive, the study of resistance relations has a long history.

Two types of resistance were identified according to two inoculation methods. In earlier times, reducing the symptom severity (GER%) was the most important objective, which became less important and has now been replaced by efforts to increase the degree of resistance to toxin accumulation. Type 1 refers to the inoculation in the middle of the ear with toothpicks infestated by the fungi or the injection of a droplet in the middle of ear for kernel resistance, and silk channel inoculation involves injecting 1–5 mL of inoculum on the top of the ear above the cob [7,8]. Early observations [9] showed that silk age influences the success of the infection. Reid et al. [10] compared different inoculation methods, with the middle ear inoculation being more effective than the silk channel inoculation. Sutton and Baliko [11] found similar results. We previously found that the silk channel inoculation less effective, and the toothpick method was chosen [12]. The main problem was that, when placing a toothpick in the silk channel, the growing cob pushed it out. The other problem was that, by injecting a given amount into the silk channel, a smaller or larger amount dripped into the soil and so an uneven level inoculum was injected into the ears. Additionally, the growing cob often grew out of the husk leaves, leading to problems in the comparison of infections [2,13]. Munkvold and White [14] concluded that the ears are mostly infected by silk mediation; therefore, the best artificial inoculation method is silk or silk channel inoculation.

As most ear rot resistance in maize is achieved by silk channel inoculation, it is necessary to explain why the toothpick method was used. A comparison of the silk channel [8,15] and the modified toothpick methods [2] demonstrated a threefold larger infection severity with the toothpick than the silk channel method, with significantly higher toxin contamination. The main problem was that the levels of infection severity between the natural and silk channel infections was nearly the same, and the toothpick method gave a 3–4-fold higher infection rate. Therefore, this method was chosen for the experimental work. Of course, we agree with Munkvold and White and Munkvold and Desjardins that most infections are mediated by silk [14,16], but for resistance tests, the kernel resistance method seems to be more feasible, at least in Hungary. In another test, the 6th day was optimal [4] and led to a significantly higher ear infection rate than the 11-day variant. Kernel resistance tests were carried out using four steel pins dipped in a macroconidial suspension of *F. graminearum* [8]. Reid and Hamilton [17] tested the silk channel inoculation technique on three maize hybrids with the same conidium concentration, and on older silks less GER severity was found. The dent forms were more resistant and produced significantly less toxins than the flint versions [18]. The authors found that the silk channel method produced a significantly lower symptom severity, with a 2–3-fold difference [18], confirming the higher infection level for the toothpick method [2,19] in terms of GER.

Since Griffing [20], the combining ability has been widely exploited in hybrid breeding to identify the best partner lines for hybrid production [21]. Moreno-Gonzales et al. [22] developed another version for statistical models, but it is not applicable to a non-diallel testing system. Previously, most papers concentrated on yield, but later, they became more focused on resistance traits, with polygenic inheritance in the background [23]. This resistance is of a polygenic nature, and authors found more QTLs in each mapping population, generally with a low or medium effect [24,25,26]. Martin et al. [26] also mapped DON contamination, which is a significant achievement beyond mainstream research. Blanc et al. (2006) [27] detected many epistatic interactions, also indicating a polygenic genetic background. Galiano-Carnerion [24], working with GER, applied a multi-parent method, evaluated in Germany and Brazil, and a stable QTL was identified across test environments, years, and locations. No toxin analysis was conducted. The prediction accuracy in the test crosses was approximately 0.50 or slightly higher. In Europe, the mean GER severity was rather uniform across test cross populations, while in Brazil, more than a fivefold difference was found. Gaikpa et al. [28] detected eight QTLs for GER, together explaining 34% of the total genetic variance. Of these, ZmSYNBREED_24070_673 was the best, with a value of 15%. Toxin data were not considered. The authors concluded that the GER resistance did not correlate with the agronomic traits tested; therefore, we believe that high resistance and good agronomy characteristics can be combined. Differences in GER resistance were also found in Hungary [12,29,30,31], but no toxin data could be attached to the GER data. The first breeding experiences were summarized in 1986 and 2000 [30,32].

For many decades, perhaps the most important question in maize breeding (and all hybrid crops) has regarded the estimation of the yield performance of inbreds in hybrids [33]. The predictions for yields ranged from 0.28 to 0.77. For now, the exploitation of the combining ability for achieving resistance has gained significance. An inheritance study [34] indicated that there is a higher chance of gaining more resistant hybrids when both inbreds have at least a medium-level resistance to *F. graminearum*. Such tests are normal in breeding programs, and even the diallel pattern allows a deeper insight. However, the toxin response was not measured.

Diallel analysis was used to test the breeding value of inbreds for hybrid production, not only for their yielding ability, but also their resistance to *Fusarium*-induced ear rot genetics. Hung and Holland [35] tested 18 inbreds for FER and found the mean hybrid vigor to be 27% for FER symptoms and a 30% lower fumonisin content, and the heterosis (combining ability) was counted by their function:

(H) = F_1_ − [(P_1_+ P_2_)/2], (H = heterosis, F_1 =_ hybrid, P_1_ = parent 1, P_2_ = parent 2)

Both the general combining ability (GCA) and specific combining ability (SCA) were significant. As the authors found higher variability in the inbreds than in the hybrids, they suggested performing a strong selection for resistance in inbreds before hybrid planning. Reid et al. [36] evaluated 11 inbreds via diallel analysis for GER resistance. The hybrid data were compared with the means of the inbred performances, as conducted by [35]. Only the resistance was analyzed by the silk channel inoculation method, and a significant GCA (general combining ability) and SCA (specific combining ability) were identified. Kernel drydown can cause pseudoresistance [37], and a common QTL was identified for the drydown and GER resistance, together supporting the idea of a pleiotropic effect [38]. As a rapid drydown decreases the ear humidity more rapidly, the disease severity also decreases, and the pathological drydown has a strong infection reduction in dry years, but not in the wet season [32]. Therefore, the pleiotropic effect is not substantiated.

Tembo et al. [39] conducted a diallel test from 12 tropical maize inbreds to measure their resistance to multiple diseases, *F. graminearum* and *Stenocarpella maydis.* The GCA indicated, that one of the 12 lines WL110-18 showed better performance against both diseases. Another diallel analysis [10] of 12 selected inbreds with differing resistance levels to *F. graminearum* by silk channel inoculation identified both GCA and SCA for the different inbreds. The most resistant line, CO272, had the largest negative GCA. Based on the GCA, the resistance performance of the hybrids could not be forecasted effectively. Responses to toxins were not added. Most genetic work has not considered toxin contamination [40]. Giorni et al. [41] identified four QTLs as effective for both *F. graminearum* and *F. verticillioides*, but neither had general resistance against the two pathogens. A rare example of the use of toxin data for genetic studies was carried out in [42], where DON and GER were similarly examined. The DON contamination was generally low, with a maximum of 7 mg/kg that seems rather low, but the GER/DON correlation was above *r* = 0.90 *(p* = 0.001). In some tests, we observed similar data, but this closeness was not true for all cases [2,3,4].

It seems that QTL analysis does not solve the combination problem with respect to resistance [43], as it does not solve the problem of breeding for higher resistance. We did not find any data that would support the validity of combining to achieve ear rot resistance in the same inbreds. The cited sources are often contradictory. As such tests did not consider toxin contamination (DON was first described in 1975 and fumonisin B1 in 1988), such a test has become possible only thereafter. After testing a high number of registered hybrids, their high variation in the GER and toxin response showed that the breeding efforts, in this respect, have only moderate success rates [2,3,4]. As the combining ability is a common problem in maize breeding, information on this subject is highly important.

In conclusion, data from the literature concentrating on the inheritance of the resistance (symptom severity) and the toxin tests are very rare, and even the legislation establishing the official limits refers to the toxin concentration without any interest in other traits. Therefore, our main objective in this study was to test the resistance to *F. graminearum* ear rot and, additionally, the resistance to toxin accumulation in order to observe how far these traits behave similarly or differently and how far we can make conclusions about toxin contamination from the infection severity data. As significant differences in the DON contamination were found for a percentage of visual infections of the inbreds and hybrids, we investigated whether a resistance background behind this phenomenon could be detected.

## 2. Results

### 2.1. GER Data

In the tests, we had 9 mother (No. G 28–G 36) and 3 father lines (No. G 37–G 39), with their 27 hybrids (G 1–G 27) and 6 control hybrids (No. G 39–G 45) from the Szeged maize breeding program. The data relating to the artificial inoculation showed significant differences in the resistance to GER (Table 1). The means of the groups for the three resistance classes of the mother lines did not differ significantly from one another. Looking at the means of the parents, the susceptible lines demonstrated the highest infection rate, but the medium- and higher-resistant mother groups achieved very similar rates. For heterosis, all groups were highly variable; the highest mean was found for the most susceptible group, the lowest for the most resistant mother group, and the medium-resistant group was in-between. An ANOVA (Table 1B) showed highly significant differences between the hybrids. Moreover, the effect of the year was very significant, and the hybrid/year interaction was significant, meaning that the positions of the hybrids showed differences in the tests. We realized that the response to natural infection of inbreds selected for their many years did have a different ranking to the artificial infection results in this test series. For this reason, the groups were newly reformed, allocating the highest infected inbreds to the susceptible group, the medium to the second group, and the most resistant to the third, moderately resistant group. Therefore, the original data are shown here, but all further conclusions for the regrouped data base will be drawn from Table 2. The ANOVA (this was not influenced by the regrouping) (Table 1B) showed highly significant differences between the hybrids. Moreover, the year effect was very significant, and the hybrid/year interaction was significant, meaning that the positions of hybrids showed differences in the tests. The 1/C and 1/D were left without further comment, and readers can see the changes that the regrouping caused. In this way, the G28 line was regrouped into the medium susceptibility group, two were transferred to the most resistant group, and one line (G34) was replaced in the susceptible group. For the past 16 years, most natural *Fusarium* infections have been recognized as being caused by *F. verticillioides*, but we know now that resistance to *F. graminearum* and *F. verticillioides* are not correlated, and the rate of *F. verticillioides* infection is normally higher than that of *F. graminearum* [2,3,4]. This justifies the transformation of the data in Table 1 into resistance groups corresponding to the artificial inoculation results (Table 2).

According to this grouping, the means of the hybrids upon artificial inoculation in the different mother groups did not differ significantly (Table 2). The means of the father and mother lines provided different results, with the highest infection severity being found for the susceptible lines, and lower severity identified for the medium-susceptible and moderately resistant groups. The mean heterosis was greatest for the susceptible and medium-susceptible mother groups (15.6% and 17.3%) and close to zero for the hybrid group with the more resistant mother lines. The mean heterosis for the three groups was 27.9%, 18.9%, and 2.4%, respectively. However, in each group, we could identify great differences in the hybrids, and the highest rate of negative heterosis (higher infection severity in the hybrids than in their parents) was found in the hybrids with the most resistant mother lines. However, looking at the original hybrid data, according to the father lines (Table 2B), two patterns can be observed: in one group, the G38 data values were higher in two cases than in the hybrids with the other two father lines, while in six cases, they were lower, and in one case, the result was in between (G32). Looking at the means of the parents (Table 1C), all G38 hybrids provided the lowest susceptibility levels compared to the G37 and G39 hybrids. This means that there is some contradiction between the data, and this has significance for the breeding strategy. Looking at the heterosis values from the 27 hybrids, 20 showed heterosis, i.e., the hybrid data were lower than the means of the parents, while in 7 cases, negative heterosis was found, concentrated in the hybrid group with the highest resistance levels of the mother inbreds.

The regression between the GER% and the variance for the eight independent data series indicated a moderately close relationship. In this case, we found genotypes with a low GER value and low variance, indicating stability. However, several hybrids were highly instable, giving very different but simultaneously similar ear rot values. For example, at 21.8% GER, the variance was only 7.4, but at the same severity, a variance above 500 was also found. Therefore, based on the mean performance, without knowing the variance, no conclusion can be drawn from the value of the given hybrid genotype and its stability.

The natural *Fusarium* infection varied significantly between 0.15% and 0.54%, and the LSD 5% was 0.11; therefore, the differences were highly significant (data not shown in detail). The means for the different isolates and years differed also significantly, being significantly higher for the natural *Fusarium* in the control (0.26%) than we observed in 2017 alone. This is 1% of the hybrid mean for the artificial inoculation. A significantly lower natural infection level was measured at Fg3 (0.17%) only in 2018, while the other data did not differ significantly from the control.

Occasionally, *Aspergillus* infection was recognized. No significant genotypic differences were found, and the mean infection severity of the control (natural infection) was 0.01%. The LSD 5% was 0.095 across isolates and years. Between the two Fg isolates, no significant difference was found for the ear infection (Fg 3 = 0.10% and Fg 4 = 0.15%).

### 2.2. DON Data

The DON contamination values (Table 3) are presented in modified order according to Table 2. DON refers to the compound, and its concentration is given in mg/kg. The DON means for the susceptibility groups showed significant differences (Table 3A). Only the mean data for the medium R group were higher than the means for the more and least resistant mother groups (59, 73, and 58 mg/kg, respectively). The means of the parents presented with less significant differences: 59, 58, and 52 mg/kg, respectively, for the S, MS, and MR groups, respectively. This indicates a slight increase in the mean resistance of the parents. In terms of the heterosis values, a −12% mean was found for the susceptible group, while for the two more resistant groups, a −28% and −34% negative heterosis was found, i.e., the increasing resistance level caused worse heterosis data. The variability within groups was significant. The highest difference was found for the R group (−198% and 63%). Of the 27 hybrids, only 10 showed positive heterosis in the F_1_ group, while the rest produced worse data than the parental means. There were four positive cases in the susceptible mother group, including one of the nine cases in the medium-resistant mother group and five in the most resistant group. This means that the hybrids had a slightly higher rate of positive cases.

The influence of the father line was variable (Table 3B,C). The hybrid data (3/B) showed that the G37 line showed the highest DON contamination among the hybrids (71.1 compared to the mother line mean of 51.58). G38 was somewhat better, with a 65.2 mean for the nine hybrids, and the lowest DON was found for G39, which was significantly better than that of G37. This line had the lowest toxin contamination even for GER (25.1 mg/kg). In terms of the combining ability of the hybrids, the medium R G38 provided only a medium DON level as the mean, but the susceptible G39 line gave the lowest mean toxin contamination. There were four genotypes with the highest DON contamination levels in the G38 hybrids (yellow highlighting). This is a good example of the specific combining ability. The rest of the data mostly correlated with the low DON contamination of the inbred G38. We do not know the reason why the low DON-producing G38 produces hybrids with a high DON contamination. Our impression is that the lines have a rather specific combining ability. For this reason, we also tested the means of the parents (Table 3C). In this case, the data correlated well with the resistance of the father lines, and the G37 and G39 hybrids had a significantly higher DON production than the G38 hybrids. It seems that, in our case, the means of the parents did not provide a robust forecasting method for the hybrids’ DON contamination levels. According to the ANOVA (Table 3D), the difference between hybrids was significant, the year effect was great and highly significant, and there was a significant interaction between years and genotypes, which did not come as was not a surprise.

### 2.3. DON Data for 1% Visual Infection

The differences in the DON production between hybrids and inbreds for a percentage of visual infection were highly significant. We experienced that, frequently, a given GER severity may be accompanied by highly differing DON contamination level. To measure this, the DON contamination value was divided by the ear rot severity, and, in this way, a number was obtained, showing the DON contamination as a percentage of infection. This is the DON%. For this reason, it became important to identify whether a detectable tendency between the inbreds and hybrids could be found (Table 4). The ear rot rate was calculated for each year and each isolate separately, and their mean values are shown in Table 4A. The ANOVA (Table 4D) was also calculated for these eight DON-producing intensity numbers. This finding means that this trait does not appear to depend on the susceptibility or resistance. The heterosis data were surprising; in the susceptible group, six hybrids showed a decrease in the DON rate and only three hybrids showed negative heterosis, i.e., a higher DON rate than that of the parents. Here, the mean heterosis was positive, with a 12.4% decrease compared to the mean of the parents. In the medium-susceptible group, the mean heterosis was −34%, with seven hybrids exhibiting negative heterosis. Only two hybrids showed a moderate heterosis lower than 10%. In the most resistant group, only one hybrid was positive, and eight hybrids showed increased DON contamination compared to the means of the parents. The negative heterosis was highest in this group, with a mean of −37.3%.

The hybrid reactions following artificial inoculation for the father lines are shown in Table 4B,D.

**Figure 1:**
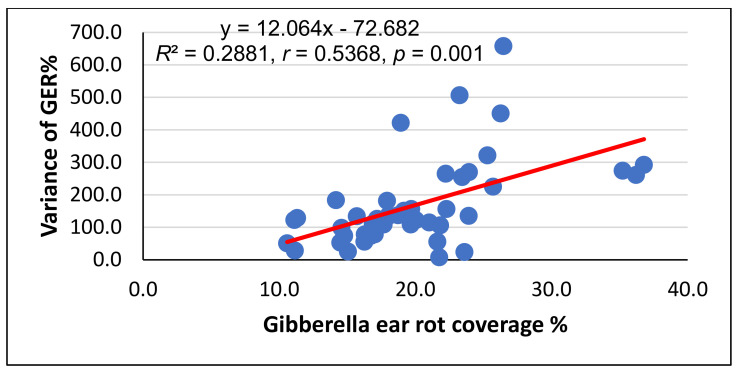
Regression between the GER and variance across eight independent databases in the GER resistance inheritance study, 2017–2020 (*n* = 45).

**Figure 2:**
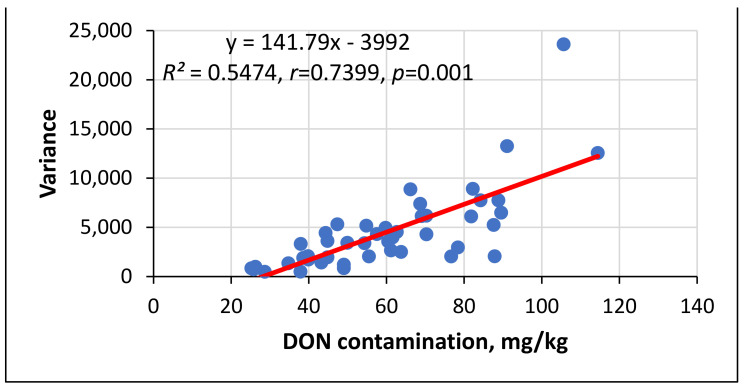
Regression between the DON contamination (mg/kg) and variance across eight independent databases in the GER resistance inheritance study, 2017–2020 (*n* = 45).

The GER data across the eight data sets showed a rather high variability, but significant differences between the individual genotypes were observed (Figure 1). The GER% and variance data correlated significantly at a medium level (*r* = 0.5368, *p* = 0.01). We observed low GER and low variance (var.) (G21: 10.6% and var. 50.6, G17: 11.1% and var. 28.5) and susceptible ones (G22: 2.8% and var. 7.4 or G1 23.6% and 23.7). On the other hand, the inbred G29 had values of 26.5% and var. 657, while hybrid 42 had a mean 14.2% and var. 183. The DON means showed unexpectedly close correlations with the variance (*r* = 0.74, *p* = 0.001). For the DON accumulation (Figure 2), two of the more resistant hybrids, including G15 and G25, produced 25 mg/kg and 26 mg/kg DON with a variance 732 and 484, respectively, and of the highly susceptible genotypes, G22 and GK3 exhibited 91.04 mg/kg and 105 mg/kg DON with a variance 13,000 and 23,000, respectively. However, this is not a genetic relation, and a high stability can also be present in highly susceptible genotypes. For us, those genotypes that have a low infection severity and low DON contamination supported by a low variance are valuable, indicating stability under different conditions and isolates.

**Figure 3:**
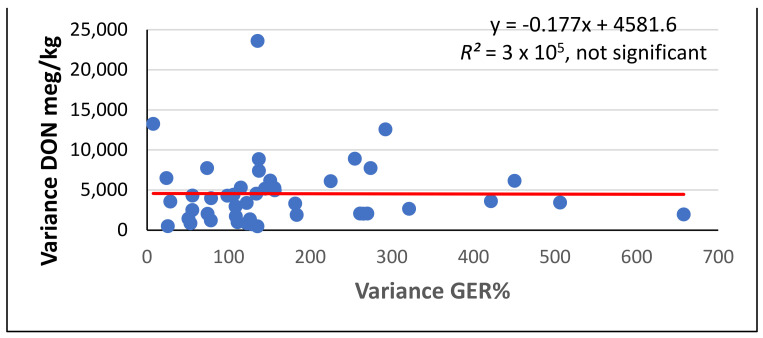
Regression between the variances for eight data series for GER% and DON (mg/kg), 2017–2020. ns = not significant.

Another method of calculation is to compare the DON contamination for a one percentage infection by comparing the rates based on the general means of the GER and DON, and not by the mean of the eight rates (data are not shown). The correlation between the two data series was *r* = 0.8881 (significant at *p* = 0.001). Fourteen hybrids were lower than average for both evaluation methods, while for six hybrids, the data did not correlate. However, among the variances between the DON and GER data, no significant correlation existed (Figure 3), as nine genotypes acted as strong correlation breakers, but for the majority, a close relationship could be proven. As nobody knows which types will have comparable data, all plans below average should be measured.

### 2.4. Comparison of the Traits and Their Heteroses and Stability

The heteroses for the different traits (Table 5) showed rather large deviations. Only two hybrids were found with positive heteroses for all traits, and five hybrids were identified with negative heteroses for all traits. We observed that, in four cases, the inbred G38 father played a role in each.

For the GER, several hybrids with only negative heterosis were found. For DON, 17 hybrids showed negative heterosis and only 10 were positive. From the correlation analysis, it appears that the GER and DON heteroses correlated positively (*r* = 0.6086, *p* = 0.01), but no significant correlations were found between these and the DON rates for a percentage of visual infection.

For the DON%, based on the ratio of the general means of GER and DON% (M/M), only three hybrids had positive heterosis. The DON% rates calculated from the eight individual cases (8) were better, and 16 negative and 11 positive heterosis rates were identified. Of the general means for the heteroses, only the GER% was positive, while others varied between −24% and −63%. We remark that all hybrids with the inbred G38 father showed a negative heterosis for all traits (five hybrids). The remaining four, for DON (mg/kg), also demonstrated a negative heterosis, but some of the other traits were positive. Looking at the rest of the hybrids, six were identified as having positive heterosis for the GER% and DON (mg/kg). In this respect, we can say that the DON% is probably regulated differently than the GER and DON data to a large extent. There was a closer correlation between the GER and DON, but they did not seem to show any commonality with the DON% responses.

In the test, considering the possible practical value of this work, the original data of the 27 hybrids were compared with the data of 6 registered hybrids (Table 6). Of these, four were identified as being equal to or lower than the means for all traits: P0216 and Sarolta (yellow hybrid name highlight). GKT376 had a DON% M/M that was only 0.01% higher than the mean; thus, it can be mentioned together with the other two hybrids. We should add that, in 2014, the P0216 had an unusually high toxin contamination with all toxigenic species. Two other hybrids (GKT 414 and GKT 376) were lower than the column mean for the DON% repl. data, and the other two control hybrids had a lower general performance. The correlations between traits, comparing the GER and DON, were *r* = 0.5433 (*p* = 0.01). The DON%, as a mean of the eight replicates, showed a less close correlation with the DON than the general means of the DON and DON%. This would support the need for further research, as this method of analysis seems to be more fruitful. Parallel with these findings, a stronger negative correlation for the GER and DON% rep. was expressed (*r* = −0.5345, *p* = 0.01) which was greater than the GER/DON% M/M correlation, which was not significant (*r* = −0.2685 ns). In Table 6, the genotypes are highlighted in green where all traits were below the mean for the given trait. Four experimental hybrids (G7, G9, G12, and G13) were identified as having a better mean for all traits. A further six hybrids were identified (including two controls) with lower GER and DON values than the mean, their names being highlighted in blue, and their performance was comparable with the best of the six commercial hybrids.

The correlations between the GER, DON, and heterosis data for the different traits (Table 7) showed that the hybrid GER% did not correlate with any other mother, father, or parental mean effects, but a significant negative relationship with heterosis was found for the GER%, DON, and DON%. This means that, generally, more susceptible reactions are connected with lower heterosis, but not without exception, of course. Significant positive correlations were found between the father and mother data and the means of the parents, indicating a stronger father effect, except for the DON%, where the mother effect was stronger. The parental mean had a significant positive correlation with all traits tested, but its practical significance is low. Therefore, the hybrid test results are more important.

We were interested in how far the agreement would hold true among variances for the GER% (mean = 169) and DON (mg/kg) (mean = 4552). According to Figure 3, a general agreement does not exist. However, we identified 15 genotypes and 1 on the line that were lower than the average values for both traits. In approximately 10 genotypes, an extreme low or high variance was detected, and for the remaining 20 genotypes, a proportional increase was found among the variances. For us, the low variances for both traits are important, as this reflects the ecological stability in terms of both resistance to disease and resistance to toxin contamination.

## 3. Discussion

The resistance data relating to natural infection were not suitable for classifying the inbreds’ resistance to *F. graminearum.* The reasons for this are that ecological conditions vary, the resistance to different *Fusarium* species mostly differs [4,13,14] and the Fusarium population largely differs [11]. The resistance to *F. graminearum* in inbred lines and hybrids cannot be determined by natural infection data. As a consequence, we used the artificial inoculation data of the *F. graminearum* isolates presented in Table 2 instead of the data based on Table 1. This ranking was used for every trait analyzed.

### 3.1. Visual Symptoms

For the visual symptoms, the groups of hybrids with differing resistant mother line groups showed non-significant differences with the same father lines. The same was true for the parental means, with a decreasing tendency towards the more resistant group, and even this was not significant. However, the variation within groups was greater and highly positive, and negative heterosis values were observed. It is remarkable that the hybrids of the MR group had the highest rate of negative heterosis of the hybrids, and the other more susceptible groups showed eight heterosis cases compared to the one negative in the group. It is therefore not an accident that the mean heterosis level was lowest in the most resistant group. The highest resistance among the father lines was measured for the G38 variant and was found to be less than half of that of the two other lines measured based on the means of the parents. However, in two cases, it was observed in the most susceptible hybrids: G30 and G34. This phenomenon is not new. Reid et al. [10] spoke of diallel tests indicating positive and negative heterosis, with the results showing that which line is the father or mother is not insignificant, as reciprocal hybrids may have different ear rot resistance levels. Previously, we found that higher resistance is more probable when both parents have higher resistance to ear rot [30]. In all other cases, the forecasting is rather unstable and may achieve different resistance levels. Applying this experiment in a setting where an entirely different set of genetic material was tested, of the G38 father and more resistant mother lines, we identified more resistant hybrids in two cases (G31, G36), and also two of the three hybrids in the moderate susceptible group. Our conclusion is that a forecasting of the resistance of hybrids is even more complicated than previously supposed. Independently from the inheritance, the higher resistance of the mother line is very useful in seed production. The conclusion is that the resistance performance of the inbred is not suitable, per se, for forecasting the resistance level of hybrids based on different combinations.

### 3.2. Toxin Evaluation

The DON data showed, in some respects, a similar picture. The means for the three maternal resistance groups and the data means for the nine hybrids did not show a clear tendency, and the middle group was more susceptible that the other two. The parental means were close to each other, with minimal difference. This situation is worse than that of the GER because, here, the majority of the hybrids produced a negative heterosis, and in all mother groups the means were negative. This means that, for many hybrids having lower GER value, we can observe a higher DON contamination. This does not generally correspond to the visual symptoms, where the situation was worse. In the father lines, G 38 demonstrated the lowest DON contamination, but their hybrids produced significantly higher DON contamination levels than the hybrids of the much more highly DON-contaminated G 39 hybrids. It seems that, genetically, we cannot forecast the toxin response based on visual symptoms. For this reason, the belief that the two traits are governed by the same genes (QTLs) is probably not true, as it could not be verified in resistance tests as valid for all genotypes [2,3,4]. For us, the reduction of the toxins is the most important consideration. In the hybrids, we can see highly significant differences between the minimum and maximum values, which enables the selection for significantly less DON contamination to be achieved. The question that remains is how we can achieve this.

### 3.3. DON Contamination and 1% Visual Infection

We found significant differences between the DON and other toxin concentrations for a percentage of visual infection (DON%) [2,3]. The differences between hybrids were also significant in this experiment, with differences of 0.80 and 4.52 mg/kg when the LSD 5% was 1.84. However, no significant difference was observed between the three mother groups. However, the differences between mother inbreds were significant across the three father lines. For this reason, some function of the genetic background can be hypothesized. We did not observe a clear-cut determining effect of the mother groups. Of the father lines, the G 37 gave the lowest mean, but in each case a significant variability was found in all three groups, similarly to the DON and GER. The means of the parents appeared to be better. The highest mean was found for the susceptible mother group (2.99 mg/kg DON for 1% GER). For the other two groups, the numbers were 1.63 and 1.45. The problem is that the hybrid reactions did not show this difference, and there should be hybrids where not only the ear rot, but also the relative toxin production, is low. It is remarkable that the highest heterosis was found in the susceptible mother group, while the others were significantly worse, and only one or two exceptions were found in these groups. This means that a higher GER%, DON contamination, and DON% are connected by unknown means with lower heterosis values. The hybrid and parental mean data showed no significant correlation. Therefore, the parental means have low significance in the breeding. This was also the case for DON and DON%, which was probably not an accident. As this is a new finding, we believe that future research will render this function more understandable. As this trait is important, we believe it will generate research to allow us to better understand disease and toxin regulation. It seems to us that this trait is important and can significantly influence the level of the toxin contamination; therefore, it should be considered in breeding and any other genetic research on resistance to toxin accumulation.

### 3.4. Interactions between Traits and Genetic Considerations

We did not find an analysis in the literature in which the GER and DON heterosis data were compared. Negative heterosis was found several times [36,44,45], and this paper supported earlier findings. Therefore, this phenomenon was not unexpected, but its extent was. In a FER/FUM diallel analysis [35], the hybrids had 27% less ear rot and 30% less FUM B1. In this respect, for the GER, we had 70% less ear rot than the parental mean, with a value for DON of 37% and for DON% of 33%. This means that, for the GER, we received better numbers, but for DON and DON%, our data were also better than those in the cited paper [35], although they were close to them. This means that a negative heterosis is not an exception and can be observed in the majority of the cases. The study published in [35] is important, as it identified poor correlations between the yield and FER, as well as FUMB1, indicating that a good combining ability for the yield does not secure the same result for the resistance or FUMB1. This shows that the situation might be similar in the case of *F. verticillioides* [3,35]. For the two versions of the DON%, the genetic background was even less clear than that found for the GER% and DON (mg/kg). Probably, the negative correlation between the original data for the traits and the heterosis, which was also valid for all traits, gives additional support for a genetic background cause.

Similar or somewhat better correlations were published when comparing the GER% and DON (mg/kg) [46], but in these tests, the maximum DON values were approximately 6 mg/kg. In our tests, the experimental mean for the DON was significantly higher. Additionally, there are even considerations that, with severe infection and high toxin contamination, the genotype differences will be smaller (over-infection). It seems to us that this is not the case, as at a higher infection severity, the differentiation of the plant population is greater [2,3,4]. It seems that the different traits are not inherited together, and while in maize we do not have example, in wheat QTLs with different functions were identified; therefore, such a situation may also be possible for maize [47].

### 3.5. Relationships between Traits

The GER/DON correlation was *r* = 0.5433, *p* = 0.05. Such data or better are normal in the literature [3,4,13,46], but mostly medium correlations have been reported. In our case, 13 hybrids gave GER and DON data that were lower than average, nine responded with data above average in both cases, and eleven produced various data, i.e., one third of the genotypes broke the correlation. Without these, the correlation was *r* = 0.8409. This shows that two-thirds of the genotypes tested here responded similarly, but one third reacted inconsequently. The strong toxin overproduction was a problem. Here, with the rate for one GER%, the DON contamination rate was significantly higher. Such was the case for G3, G5, or G11, and GKT3275. The DON% data correlated negatively with GER%, indicating that a lower GER infection resulted in a higher probability of producing a higher DON% (*r* = −0.5345, *p* = 0.01), but with the DON% M/M being less negative. However, the DON contamination correlated positively with the DON (*r* = 0.6405); therefore, this trait seems to be better for the serial work. When we examine all three traits, 3 hybrids were found with all traits classified as susceptible, 4 showed lower data for all traits than the mean, and 16 varied.

When we examine only the hybrids that gave similar results for the GER, DON, and DON% M/M, 13 reacted uniformly for the 3 traits, 7 were susceptible for all, and 6 had values lower than the mean for all traits. This is better than the nine hybrids that, for all traits, presented with uniformly high or low resistance.

Adaptation ability is an important trait for GER resistance behavior. This can be measured by the variance, calculated by the one-way ANOVA conducted using the MS Excel program. Among the inbreds, three major reaction types could be identified. The first group was characterized with a proportional variance for both the GER% and DON (mg/kg), ranging from a low to high mean performance. We require genotypes with a low variance for GER, DON, and DON% M/M. Therefore, in addition to the low value, it is better when a hybrid has a 1% GER at a variation of 20 than the same mean with a variation of 200, indicating that the former hybrid will probably have low infection and low DON rates across different ecological regimes. This is not surprising for plant breeding, where the yield stability is one of the most important traits. We add to this that the same principle is similarly important in matters of resistance to toxigenic fungi. This work shows that such hybrids can be identified. The problem is that this requires a far more extended database, as from two or three data sets such an analysis has only very minimal value.

Of course, in the DON, other genetic and ecological conditions may play a role, as the two isolates in the four years provided a mean (*n* = 45) whereby the mean DON contamination varied between 21 and 184 mg/kg, and the two isolates gave a performance of 85 and 63 mg/kg across years. The two isolates behaved as independent units, and the correlations were not closer between them than they were between the other isolates in different years. Thus, we had eight different-looking data series. Between the eight data series, only three significant correlations were found among the 21 hybrids. This means that even the same isolates produced different results in different years. In other words, one inoculum in 2–3 years will hardly produce data of an adequate quality to properly evaluate the resistance and toxin response. The GER data showed better cohesion. The use of more isolates [2,3,4,12] proved useful previously and can double or triple the data set available for more exact phenotyping. Here, 9 significant correlations were identified, while 12 were not significant. The data clearly show that the genetic regulation differed between the GER and DON and the DON% data, where diverging genotype groups could be identified. It seems that a significant part of the hybrids behaved similarly across the traits tested, but the larger rate showed highly variable results, with probable different genetic backgrounds [2,3,4,12]. We should mention that similar problems were faced by researchers working with *F. verticillioides* and *A. flavus*, and it will not be easy to combine them [12]. The task is how to identify inbreds that have a good combining ability to protect against all three ear rot-causing agents. As the findings from resistance studies are encouraging [3,48,49], and this paper also indicates the possibility of identifying such genotypes, progress in this very complicated field is possible.

### 3.6. Breeding Aspects

Heterosis is a key element of the breeding of hybrid crops. Originally, it was understood the yielding ability and the general and specific combining abilities (GCA and SCA) were distinguished. The first designated inbreds demonstrated a significant positive heterosis for most of the other inbreds, and the second was applied to inbreds that provided some cases of high hybrid vigor, but mostly did not. The idea that a combining ability (heterosis) may also exist in corn diseases is not new. Reid et al. [10] published a paper based on diallel analysis and proved its usefulness in genetic research pertaining to GER resistance. The significance of breeding resistance and decreasing toxin contamination is stressed, but toxin data are seldom published. This study showed that heteroses for disease symptoms and toxin reduction are not interchangeable traits. Whereas a disease reduction was found in the majority of the hybrids compared to the parents, for the DON, the majority of the hybrids showed negative heterosis, i.e., the DON contamination of the hybrid was higher than that of the parental means.

As heterosis varies with different traits and a low DON contamination is required, a selection system should be built that can fulfil these requirements. In a recent paper [3], it was suggested that the selection should not be initiated with inbred lines for two reasons. The first is that heterosis outcomes for the yield and GER will not agree necessarily. In this way, the screening of inbreds for GER does not automatically secure a high yielding ability with a low DON contamination and low DON% for a percentage of visual infection. On the other hand, resistance to GER will not automatically provide resistance to *F. verticillioides* and *A. flavus,* as was proven for hybrids [2,3,4,12,49,50].

It does not seem to be reasonable to screen hundreds of inbreds for the three traits tested in a breeding program, as more than a hundred thousand inoculated heads are required. The data do not, per se, provide a good forecast of hybrid vigor in terms of the yield and resistance with the different traits. Including the other two important ear rot pathogens would triple the amount of work. Instead, we should screen the experimental hybrids of the breeding program whose inbreds are present in the gene pool. For resistance testing only, those hybrids whose yielding ability is above the control limit should be tested. The testing system in [3] shows the details. By analyzing the parental structure of the inbreds, those that participate in more than one hybrid can be identified. This information can help to plan new combinations with the necessary yield and resistance traits, and it is much cheaper than screening hundreds of inbreds without knowing anything about their heterosis outcomes beyond the yielding ability of different traits. Now, we see this as a chance to develop better hybrids in a short time and to withdraw highly susceptible hybrids from commercial production. Parallel with these efforts, a breeding program should also be started that allows more resistant inbreds with superior heterosis in the requested traits to be produced. Care should be taken to secure good or high resistance to FER and AER in the mother and/or father lines, as in terms of the seed production, this is an important prerequisite, and the variability of the inbreds allows for this to be achieved [48]. Careful screening is important, as a significant portion of hybrids have higher or lower contamination degrees than those forecasted by the performance of the parents. It is encouraging that the FER resistance and yield demonstrated no strong relationship [49]; therefore, breeding methods for a high resistance and yield can support one another, and this could also be the case for GER. We had the same experience in previous work [3]. American data [50] support the view that heterosis and resistance to FER can be independent phenomena. When both parents were better in terms of their FER resistance, resulting hybrids with a good combining ability for the yield were identified, with an SCA for the FER. The FUM was not measured; thus, the food safety aspects could not be analyzed. In 13 of the 21 hybrids tested, negative heterosis was found for the FER. The same was found for the GER in this paper and that of Reid et al. [10]. Additionally, Fan et al. [23] verified the results of Reid et al. [10], showing that reciprocal crosses have a great impact on heterosis expression, so that a general conclusion cannot be drawn from a single hybrid test for the reciprocal variants. For the grain yield, however, all hybrids showed positive heterosis.

Of the six control hybrids, three hybrids were superior to the mean performance, while PO216 and Sarolta presented with data below the means for all traits. Additionally, GKT 414 and GKT 376 could be grouped together, as they had three positive data results. Of the experimental hybrids, G 7, G 9, G 12, and G 23 belonged to this group. This means that, from the 27 experimental hybrids, 4 surpassed the resistance of the superior commercial control hybrids. Three hybrids were identified with a consequent susceptibility to all traits; and 20 gave variable responses.

A number of QTL analyses of the ear rot pathogens of maize have been conducted [13]. However, to date, no resistance genes have been cloned, and only several QTLs were validated. Therefore, the vast majority of the inbred and hybrid production methods cannot use this technology. For this reason, more precise screening methods are important in order to identify better hybrids and inbreds. As our knowledge of genetics increases, their impacts may be utilized in the future.

### 3.7. Methodical Conclusions

The use of two independent isolates made it possible to achieve a much wider database for the stability analysis of resistance traits. For testing using two years’ worth of data with one inoculum (pure isolate or mixture), such an analysis is not possible; even growers would require such data. It also became clear that, alone, GER data cannot provide a reliable picture of the food safety risks of hybrids or inbred lines and do not reveal anything about the toxin production at a 1% infection severity. Recent data support this observation [2,3]. Experiments forming the basis for the introduction of an updated variety registration methodology were published [2,3,4], and its introduction was recommended as soon as possible. As a maize registration test lasts two years (when disagreement is involved, it is three), we increased the suggested number of isolates to three in two locations. In this way, the tests are reliable enough to make conclusions regarding their usefulness. This relates not only to the qualification of the hybrids, but also to phenotyping in genetic and molecular genetic studies, where this can increase the reliability of the QTLs identified. It also appears that a reasonably high ear rot severity and toxin contamination are needed to better differentiate the genotypes tested.

## 4. Conclusions

The inheritance of resistance to GER is much more complicated than supposed previously [51]. This should be taken into consideration in future tests. It is possible to select hybrids with a low GER and DON contamination, which we believed would be sufficient until now, but this area of research requires more work. As the performance of hybrids does not often follow the resistance of inbreds, the tests do not, per se, guarantee a good hybrid performance. As the host–pathogen relationship is very sensitive, this can be balanced by wider testing to gain more data in order to ensure a solid decision. In maize, GER is not the only problem. Thus, for successful breeding, *Fusarium* ear rot (FER) and *Aspergillus* ear rot (AER) should also be considered in order to secure a low risk for all important ear rot pathogens in regions where all these pathogens occur and cause heavy losses in epidemic years. Extensive serial screening work is highly important for detecting unknown resistances in breeding programs, and as databases grow, we will have more powerful data set to support breeding in order to increase food safety without decreasing the yield.

## 5. Materials and Methods

### 5.1. Plant Material

Experimental hybrids were processed by top crosses to avoid the need for hand pollination to produce hybrid seeds over a four-year period. Inbreds from Cereal Research Ltd. (Szeged, Hungary) were selected for this work based on observations of their infection history over the past years. Thus, the uniformity of the hybrids could be secured for the whole experiment (*n* = 27). According to previous observations regarding the inbreds, three susceptible (G 39, G 30, G 34), three medium susceptible (G 28, G 33, G 35), and three moderate resistant inbreds (G31, G 32, G 36) (*n* = 9) were chosen based on earlier natural infection observations of the mother lines. This is not to say that the latter had immunity. They were crossed by three father lines (*n* = 3) with differing resistance levels based on observations of natural infection, so that the effects for all mothers and fathers and their means could be presented. G 37 was susceptible, G 38 was moderately resistant, and 39 was moderately susceptible. Altogether, 45 genotypes were tested, with 27 experimental hybrids, 9 mother and 3 father lines, and 2 commercial hybrids with different resistance levels, which were included to enable the classification of the resistance or susceptibility of the experimental hybrids.

Each plot consisted of two rows that were 5 m long (75 cm rows and 20 cm plant spacing). In a row, approximately 20 plants were inoculated by the modified toothpick method [2,4]. Each treatment was performed in two replicates and two independent isolates in a randomized block design. Thus, altogether, 8 independent data series with 16 data categories stood behind each mean hybrid datum between 2017 and 2020.

### 5.2. Experimental Conditions and Design

The tests were conducted by Cereal Research nonprofit Ltd., Szeged, Hungary, Exp. Station Kiszombor, in the Maros valley, 25 km east of Szeged (GPS coordinates: 46°12′49.0″ N and 20°09′57.9″ E). The soil here has a high clay content that enables tillage when draught is predominant. The precipitation varies between 350 and 1100 mm a year, while the soil pH is neutral (6.98). The latest humus content is 2.21%. N is very low (5.8 mg/kg), P_2_O_5_ is 280 mg/kg, K_2_O is 317 mg/kg, and Mg is 376 mg/kg. The Zn and SO_4_ ion concentrations are low. In autumn, 160 kg of Genesis complex fertilizer was added, while in spring, 80 kg/ha was added (nitrosol; 46% carbamide), both from Pét Nitrocomplex Ltd., Pétfürdő, Hungary. Irrigation was performed after sowing (between 25 April and 5 May), where it was necessary to reach a uniform germination. The middle of June and the middle July were the next necessary irrigation times. Decis (0.2 L/ha) was used to control the corn borer (Bayer Hungaria Ltd, 1117 Budapest, Hungary, Dombovari str. 26,affiliated firm of Bayer Inc., Leverkusen, Germany) a.i., 50 g/L of deltamethrin). Weed control was achieved using 4.5 L/ha of Lumex (375 g/L of mesotron, 375.0 g/L of S-metolachlor, and 125.0 g/L of terbutaline), Desormon (375 g/L of mesotron and 375 g/L of S-metachlor from Nufarm Hungaria Ltd.), or Shadow 200 (200 g/L of dimethenamid-P, 200 g/L of metazachlor, and 100 g/L of quinmerac) from BASF Agr. Solutions (https://www.agricentre.basf.co.uk/en/About-us.html, U.K., Head Quarter: BASF Ludwigshafen, Carl-Bosch-Straße 38, Germany, accessed on 24 August 2022) at a rate of 2.5 L/ha, depending on the composition of the weeds.

The more important meteorological data showed some differences in temperature (Table 8). In June, the precipitation was generally high, except in 2017, while July was moderate in this year but high in 2020. August was higher than usual in 2018–2020, and October was very high again. The regular yearly precipitation is normally 550–600 mm in a year, with very high variability between years within each season. Therefore, after sowing, 30–40 mm irrigation was applied when necessary. The next possible irrigation times were the middle of June, the end of July, and the beginning of August.

### 5.3. Isolates and Inoculation

The *F. graminearum* strains were isolated from naturally infected Hungarian maize grain samples. Their monosporic lines were identified by the IGS-RFLP method [52] . Two *F. graminearum* isolates (Fg3 and Fg4) were used, which were selected based on their aggressiveness and DON production and had somewhat differing levels of aggressiveness. The strains were deposited in the Microbe Gene Bank of Cereal Research Nonprofit Ltd., which is part of the Hungarian National Centre for Plant Diversity and is freely accessible. Their deposit numbers were as follows: Fg3: NGBAB142629; Fg4: NGBAB142696. To identify the strains, the PCR marker, EF1-α primer ef1 ATGGGTAAGGARGACAAGAC, was used. From the beginning of silking, every second day, the number of silking plants was recorded. The inoculation time was six to seven days after mid-silking. The preparation of the infestated toothpicks was conducted following the concept of Young [53] (it was published in a short abstract in *Phytopathology* without methodical details), modified by that of Mesterházy et al. [3,4,12]. In the middle of the upper ear (only these were inoculated), a hole was made using an awl, being 15 mm long and 1.5 mm wide, and an infested toothpick was placed in this hole, where it remained until harvesting. As the spreading of the infection stopped at 23% grain moisture on the ear surface, leaving earlier hybrids for longer did not cause a problem [54].

When the last hybrid ripened and the plants were dried, the ears were harvested. The water content of the grains was 18% or lower, as measured by TwistGrain Pro. (Draminski Electronic in Agriculture, www.draminski.com, Draminski S.A., Wiktora Steffena 21, Sząbruk, 11-036 Gietrzwałd, Poland, accessed on 12 July 2022). For the evaluation, only those ears were considered that had toothpicks, as their fungal infection or the trace of the toothpick in the ear could be identified. Insect-damaged ears were not considered; those ears were discarded from further evaluation. In this way, the influence of insect damage and resulting additional toxin contamination could be excluded from the evaluation and the probability increased significantly that the differences observed were really differences in resistance. After the evaluation, five ears with medium severity were separated in a Rashed bag and delivered to a dry room within 24–28 h after harvest. Stalk rot was typically not found, and premature death was also not recognized. This is important, as stalk rot causes a pathological drydown that can reduce ear rot even by 50% [29], thereby causing pseudoresistance. Therefore, a lower plant density (66,000 plants/ha) and irrigation helped to inhibit premature death. The resistance of the hybrids to stalk rot was much higher than it was 50 years ago [30], and this also helped us to achieve more reliable ear rot and toxin data. For the heterosis evaluation, the means of the father and mother were compared to the performance of their hybrids [55,56].

### 5.4. Evaluation of Symptoms and Deoxynivalenol

**Figure 4:**
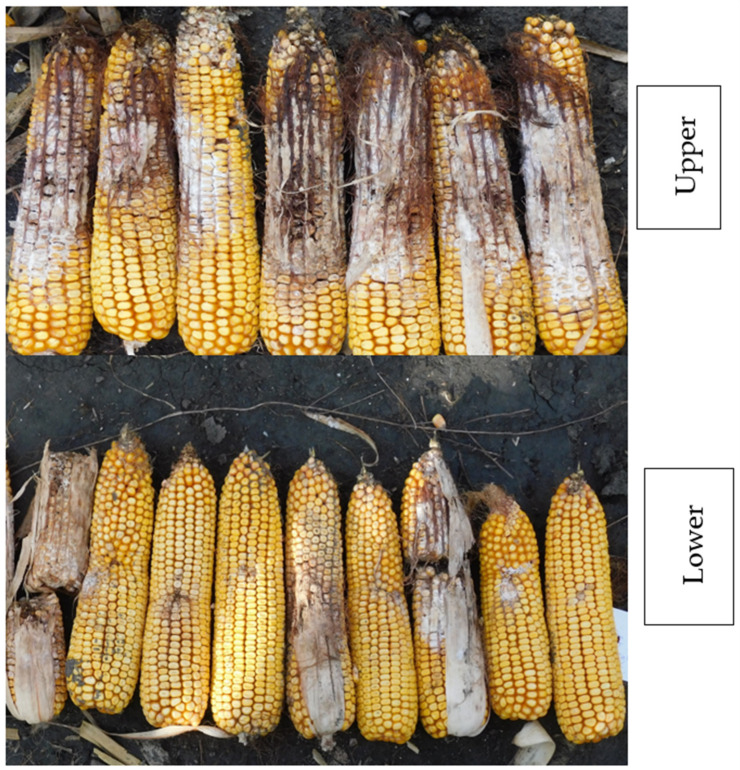
**Upper**: susceptible hybrid, with 15–60% variation in infection severity; **right**: a mor resistant hybrid. **Lower**: more resistant, some ears have only 1–2 grains infected, and the track of the toothpicks can also be seen.

For the evaluation, the scale suggested by Reid et al. [8] was developed further, as often the disease severity of *F. verticillioides* and *A. flavus* are rather low and therefore needed updating [3,4]. The scale [56] for *F. graminearum* classifies the symptomless ears as category 1, while all ears having a lower severity than 3% are classified in the 2nd category. The 3rd category includes ears with an infection severity between 4–10%, class 4 considers ears have 11–25% ear rot severity, class 5 have 26–50%, class 6 have 51–75%, and class 7 have 76–100%. This method is not sensitive enough to identify a relation with DON, as in some tests, even the highest number is in the 2nd class. The scale identifies the visually infected kernel rate on the unshelled maize ears. For less aggressive isolates, a more sensitive scale was needed. As we wanted to compare the visual symptoms with the toxin contamination, a significant refining of the scale was also needed. For the artificial inoculation, the amount of infection around the toothpick was rated. Natural infection caused by *Fusarium* spp. was also evaluated. Those infected areas (with a white or rose discoloration) were evaluated here and were seemingly independent from the infection caused by the toothpick on other parts of the ear. In several cases, *Aspergillus* spp. also caused natural infection, with light to deep green discoloration. The scale used for the evaluation ranged from 0% to 100%. A similar outcome was reported by Reid et al. [57], but the resolution was much higher, as described [2]. Generally, 1 ear contains about 700–800 grains. When one grain is infected, this is about 0.15%. For 7–8 grains, it means 1%. For 15 grains, it means 2%. This detailed evaluation continues up to 5% and, after 10%, each class is 10% higher, continuing up to 100%. The natural infection on the artificially inoculated ears was normally much lower than 1% of the ear surface. Of the ears in a row, 10–15 could be evaluated, and in several cases this number could be lower than ten. The reason for this is that, of the plants, not all could be inoculated, as in the case of late-flowering small plants. We could identify as infected only the plants where the track of the toothpick was clear; otherwise, a zero number could not be rated. The means for a row served as the entries into the ANOVA. In a parallel test with other hybrids on the same field, the natural infection was measured, showing a natural ear infection between 2017 and 2018 ranging from 0.12–0.68%, as well as 0.05–0.30% for the *Fusarium spp*. infection severity between 2019 and 2020. The natural *Fusarium* and *Aspergillus* spp. data were available. However, the Fusarium natural infection was caused mostly by *F. verticillioides* or *A. flavus*, which did not have common toxins with *F. graminearum*. They were not analyzed in detail, and no toxin analysis was conducted on them for the same reason. After evaluation, five ears with an average infection severity were selected for toxin measurement. After harvest, the evaluation was performed, and within 24 h, the sample ears were under a roof in a dry room in the laboratory. Figure 4 shows the different genotypes and the variation within a genotype.

For the DON%, two calculations were conducted. The DON% rates were calculated for each of the eight data sets, and their means are presented in Table 4. Another method was to count the means of the eight data sets and compare their means. This was designated as DON% M/M (DON mean/GER mean), as presented in Table 6, in the last column.

Following a two-week drying period of the five ear samples, the ears were shelled. After shelling, the whole amount (approximately 1 kg) was roughly ground into 1–2 mm particles in order to obtain a much better distribution of the DON in the ground material compared to the whole grain sample. In this way, the sampling error could be significantly reduced [3,4]. This sample was mixed thoroughly, and 100 g of the ground sample was separated. The same preparation method was used in the other replicate. The two 100 g samples were pooled and mixed together. From this, 50 g was separated and given to the analytical lab. This was finely milled to powder using the Perten Laboratory Mill (Type: 3310, Perten Instruments, 126 53 Hagersten, Sweden). The DON was measured by an Agilent Infinity 1260 HPLC (Agilent, Santa Clara, CA, USA), using the method described by Szabó et al. [4].

### 5.5. Statistical Methods

As the framework of the diallel analysis [20] could not be applied, for the evaluation of the data, three-way ANOVAs were conducted using the Excel function for the visual symptoms and deoxynivalenol data. The rates for the DON contamination for a 1% visual infection severity were produced for all the hybrids and years, and they were subjected to ANOVA to identify whether the traits tended to have a genetic background or not. First, a two-way analysis was conducted, which produced all of the totals for the replicates, and with these sums, a three-way AN0VA was carried out using the functions described by Sváb [58] and Weber [59]. From the test, we obtained the MQ values, and these were used to present the LSD 5% data for the hybrids and hybrid groups. Heterosis was calculated by comparing the parental means of the inbreds with the performance of the hybrids. Positive hybrid heterosis was found when the performance of the hybrids was lower than the mean of the parents. Negative heterosis could be identified when the hybrid was infected more than the mean of the two parental lines.

## Figures and Tables

**Table 1:** Combining the ability of inbreds of maize with *F. graminearum* ear rot (%), with the ear rot coverage as a percentage, and resistance classification based on responses of inbreds to natural contamination, 2017–2020.

(**A**) Ear rot coverage caused by *F. graminearum* as a percentage, and resistance classification based on responses of inbreds to natural contamination												
**Hybrid**	**Mother**	**Father**	**Mother Group**	**Hybrid Fact %**	**Mean of Group**	**Mother Fact**	**Father Fact**	**Mean of M + F**	**Mean of Group**	**Heterosis %**	**Mean Heteroses of Groups %**	
	Line	Line		Mean		%	%	%			%	
G 1	28	37	S. inbreds	23.60		20.03	36.20	28.12		16.0 *		
G 2		38		16.88		20.03	11.11	15.57		−8.4		
G 3		39		18.69		20.03	26.27	23.15		19.2		
G 4	29	37		23.43		26.47	36.20	31.34		25.2		
G 5		38		16.72		26.47	11.11	18.79		11.0		
G 6		39		25.92		26.47	26.27	26.37		1.7		
G 7	30	37		17.67		35.22	36.20	35.71		50.5		
G 8		38		25.70		35.22	11.11	23.17		−10.9		
G 9		39		15.05	**20.4**	35.22	26.27	30.74	**25.9**	51.1	**17.3**	
G 10	31	37	MR/MS inbreds	19.70		17.18	36.20	26.69		26.2		
G 11		38		14.57		17.18	11.11	14.15		−3.0		
G 12		39		18.54		17.18	26.27	21.73		14.6		
G 13	32	37		36.78		17.49	36.20	26.85		−37.0		
G 14		38		22.23		17.49	11.11	14.30		−55.4		
G 15		39		11.31		17.49	26.27	21.88		48.3		
G 16	33	37		19.67		21.62	36.20	28.91		32.0		
G 17		38		11.15		21.62	11.11	16.36		31.9		
G 18		39		19.17	**19.2**	21.62	26.27	23.94	**21.6**	19.9	**8.6**	
G 19	34	37	MR. Inbreds	14.77		23.93	36.20	30.07		50.9		
G 20		38		22.44		23.93	11.11	17.52		−28.1		
G 21		39		10.58		23.93	26.27	25.10		57.9		
G 22	35	37		21.75		21.64	36.20	28.92		24.8		
G 23		38		16.28		21.64	11.11	16.38		0.6		
G 24		39		19.25		21.64	26.27	23.96		19.6		
G 25	36	37		23.92		18.73	36.20	27.47		12.9		
G 26		38		15.70		18.73	11.11	14.92		−5.2		
G 27		39		23.24	**18.66**	18.73	26.27	22.50	**22.98**	−3.3	**14.4**	
Mean				19.44	19.4	22.48	24.53	23.50	23.5	13.44	13.4	
LSD 5%				5.64	1.90							
* Yellow highlighting: useful positive heterosis.												
(**B**) ANOVA												
**Source of Var.**		**SS**		**df**		**MS**		**F**			**LSD 5%**	
Hybrid A		25,196.97		44		572.7		8.67		***	5.64	
Year B		56,905.94		3		18,968.6		287.27		***		
Isolates C		39.19		1		39.2		0.59		ns		
A × B		34,896.91		132		264.4		4.00		***		
A × C		3118.19		44		70.9		1.07		ns		
B × C		2507.19		3		835.7		12.66		***		
A × B × C		9532.06		132		72.2		1.09		ns		
Within		23.88		360		66.3						
Total		155,078.9		719								
*** *p* = 0.001, ns = not significant												
(**C**) Hybrid performance of the inheritance tests of GER depending on the performance of the inbreds of the mother and father inbreds.												
**Inbred**	**Mother**	**G 28**	**G 29**	**G 30**	**G 31**	**G 32**	**G 33**	**G 34**	**G 35**	**G 36**	**Mean**	**LSD**
Father	per se	20.03	26.47	35.22	17.18	17.49	21.62	23.93	21.64	18.73	22.48	5%
G 37	36.20	23.60 *	23.43	17.67	19.70	36.78	19.67	14.77	21.75	23.92	**22.37**	
G 38	11.11	16.88	16.72	25.70	14.57	22.23	11.15	22.44	16.28	15.70	**17.96**	
G 39	26.27	18.69	25.92	15.05	18.54	11.31	19.17	10.58	19.25	23.24	**17.97**	
Mean		**19.73**	**22.02**	**19.48**	**17.60**	**23.44**	**16.66**	**15.93**	**19.10**	**20.95**	**19.44**	**3.25**
Mother group				20.41			19.24			18.88		**1.88**
Father lines												**1.45**
* Yellow highlighting: strong difference between the resistance of the father line and the hybrid.												
(**D**) Means of the father and mother lines in the inheritance tests of GER depending on the performance of the inbreds of the mother and father lines.												
**Inbred**	**Mother**	**G 28**	**G 29**	**G 30**	**G 31**	**G 32**	**G 33**	**G 34**	**G 35**	**G 36**	**Mean**	
Father	per se	20.03	26.47	35.22	17.18	17.49	21.62	23.93	21.64	18.73	22.48	
G 37	36.20	28.12	31.34	35.71	26.69	26.85	28.91	30.07	28.92	27.47	29.34	
G 38	11.11	15.57	18.79	23.17	14.15	14.30	16.36	17.52	16.38	14.92	16.80	
G 39	26.27	23.15	26.37	30.74	21.73	21.88	23.94	25.10	23.96	22.50	24.37	
Mean		**22.28**	**25.50**	**29.87**	**20.86**	**21.01**	**23.07**	**24.23**	**23.09**	**21.63**	**23.50**	
				25.88			21.65			22.98		
The hybrid performance and parental means often disagreed, especially in terms of the hybrid performance (Table 1C,D), bold data: means of hybrids across father lines.												

**Table 2:** Combining the ability of the inbreds of maize with *F. graminearum* ear rot (%), with the ear rot coverage as a percentage, and the resistance classification based on the responses of mother lines according to their artificial inoculation data, 2017–2020.

(**A**) Ear rot coverage caused by *F. graminearum* as a percentage, and resistance classification based on responses of inbreds to artificial inoculation												
**Hybrid**	**Mother**	**Father**	**Mother Group**	**Hybrid Fact %**	**Mean of Group**	**Mother Fact**	**Father Fact**	**Mean of Parents**	**Mean of Group**	**Heterosis %**	**Mean of Heterosis %**	**Change of Heterosis**
	Line	Line		Mean		**%**	**%**	**%**			%	%
G19	34	37	S inbreds	14.77		23.93	36.20	30.07		20.5 *		
G20		38		22.44		23.93	11.11	17.52		10.4		
G21		39		10.58		23.93	26.27	25.10		7.4		
G7	30	37		17.67		35.22	36.20	35.71		−3.0		
G8		38		25.70		35.22	11.11	23.17		4.0		
G9		39		15.05		35.22	26.27	30.74		63.2		
G4	29	37		23.43		26.47	36.20	31.34		25.2		
G5		38		16.72		26.47	11.11	18.79		11.0		
G6		39		25.92	**19.14**	26.47	26.27	26.37	**26.53**	1.7	**15.60**	27.9
G1	28	37	MR/MS inbreds	23.60		20.03	36.20	28.12		16.0		
G2		38		16.88		20.03	11.11	15.57		−8.4		
G3		39		18.69		20.03	26.27	23.15		19.2		
G16	33	37		19.67		21.62	36.20	28.91		32.0		
G17		38		11.15		21.62	11.11	16.36		31.9		
G18		39		19.17		21.62	26.27	23.94		19.9		
G22	35	37		21.75		21.64	36.20	28.92		24.8		
G23		38		16.28		21.64	11.11	16.38		0.6		
G24		39		19.25	**18.5**	21.64	26.27	23.95	**22.8**	19.6	**17.3**	18.9
G10	31	37	MR Inbreds	19.70		17.18	36.20	26.69		26.2		
G11		38		14.57		17.18	11.11	14.15		−3.0		
G12		39		18.54		17.18	26.27	21.73		14.6		
G13	32	37		36.78		17.49	36.20	26.85		−37.0		
G14		38		22.23		17.49	11.11	14.30		−55.4		
G15		39		11.31		17.49	26.27	21.88		48.3		
G25	36	37		23.92		18.73	36.20	27.47		12.9		
G26		38		15.70		18.73	11.11	14.92		−5.2		
yes		39		23.24	**20.7**	18.73	26.27	22.50	**21.2**	−3.3	**−0.2**	2.4
Mean				**19.44**	6.9	**5.93**	**8.18**	7.06	7.1	**−0.07**	−0.1	0.8
LSD 5%				5.60	1.88	3.25	1.45					
* Yellow highlighting: useful positive heterosis.												
(**B**) Hybrid resistance data (bold) from the GER inheritance study, with ear rot data as percentages.												
**Inbred**	**Mother**	**G 34**	**G 30**	**G 29**	**G 28**	**G 33**	**G 35**	**G 31**	**G 32**	**G 36**	**Mean**	**LSD 5%**
Father	per se	23.93	35.22	26.47	20.03	21.62	21.64	17.18	17.49	18.73	22.48	
G 37	36.20	14.77 *	**17.67**	23.43 **	**23.60**	**19.67**	**21.75**	**19.70**	**36.78**	**23.92**	**22.37**	
G 38	11.11	**22.44**	**25.70**	**16.72**	**16.88**	**11.15**	**16.28**	**14.57**	**22.23**	**15.70**	**17.96**	
G 39	26.27	**10.58**	**15.05**	**25.92**	**18.69**	**19.17**	**19.25**	**18.54**	**11.31**	**23.24**	**17.97**	
Mean	24.52	**15.93**	**19.48**	**22.02**	**19.73**	**16.66**	**19.10**	**17.60**	**23.44**	**20.95**	**19.44**	3.25
Mean	Mother groups			19.14			18.50			20.67		1.88
* Yellow highlighting: unexpected hybrid reactions compared to the parental means (Table 2**C**), ** Expected hybrid data.												
(**C**) Hybrid resistance data (bold) from the GER inheritance study, parental means, with ear rot data as percentages.												
**Inbred**	**Mother**	**G 34**	**G 30**	**G 29**	**G 28**	**G 33**	**G 35**	**G 31**	**G 32**	**G 36**	**Mean**	
Father	per se	23.93	35.22	26.47	20.03	21.62	21.64	17.18	17.49	18.73	22.48	
G 37	36.20	30.07 *	**35.71**	**31.34**	**28.12**	**28.91**	**28.92**	**26.69**	**26.85**	**27.47**	**29.34**	
G 38	11.11	**17.52**	**23.17**	**18.79**	**15.57**	**16.36**	**16.38**	**14.15**	**14.30**	**14.92**	**16.80**	
G 39	26.27	**25.10**	**30.74**	**26.37**	**23.15**	**23.94**	**23.96**	**21.73**	**21.88**	**22.50**	**24.37**	
Mean	24.52	**24.23**	**29.87**	**25.50**	**22.28**	**23.07**	**23.09**	**20.86**	**21.01**	**21.63**	**23.50**	
Group mean				26.53			22.81			21.17		

* Bold: Hybrid performance and their means across father lines.

**Table 3:** Combining ability of the inbreds of maize for *F. graminearum* ear rot and DON contamination (mg/kg) in 2017–2020, with the resistance classification based on the responses of mother lines, according to their artificial inoculation data, 2017–2020.

(**A**) DON data of the hybrids, mg/kg												
**A/Hybrid**	**Mother**	**Father**	**Mother Group**	**Hybrid Fact %**	**Group Mean**	**Mother Fact %**	**Father Fact %**	**(M + F)/2 Mean**	**Group Mean**	**Heterosis %**	**Heterosis Mean**	**Heterosis Change**
	Inbred			Mean		**%**	**%**	**%**			%	%
G 19	M34	F37	S inbreds	55.50		39.80	87.87	63.84		13.1 *		
G 20		F38		44.33		39.80	25.21	32.50		−36.4		
G 21		F39		43.24		39.80	69.08	54.44		20.6		
G 7	M30	F37		39.88		88.83	87.87	88.35		54.9		
G 8		F38		81.82		88.83	25.21	57.02		−43.5		
G 9		F39		37.85		88.83	69.08	78.96		52.1		
G 4	M29	F37		82.23		44.80	87.87	66.33		−24.0		
G 5		F38		84.24		44.80	25.21	35.00		−140.7		
G 6		F39		61.17	**58.9**	44.80	69.08	56.94	**59.3**	−7.4	**−12.4**	0.67
G 1	M28	F37	MR/MS inbreds	89.55		54.33	87.87	71.10		−26.0		
G 2		F38		57.51		54.33	25.21	39.77		−44.6		
G 3		F39		105.62		54.33	69.08	61.70		−71.2		
G 22	M35	F37		91.04		47.35	87.87	67.61		−34.7		
G 23		F38		49.01		47.35	25.21	36.28		−35.1		
G 24		F39		54.82		47.35	69.08	58.22		5.8		
G 16	M33	F37		78.42		63.76	87.87	75.81		−3.4		
G 17		F38		60.46		63.76	25.21	44.48		−35.9		
G 18		F39		70.27	**73.0**	63.76	69.08	66.42	**57.9**	−5.8	**–27.9**	−26.08
G 10	M31	F37	MR inbreds	59.77		34.75	87.87	61.31		2.5		
G 11		F38		70.31		34.75	25.21	29.98		−134.5		
G 12		F39		37.91		34.75	69.08	51.91		27.0		
G 13	M32	F37		114.42		26.24	87.87	57.06		−100.5		
G 14		F38		76.66		26.24	25.21	25.73		−198.0		
G 15		F39		25.76		26.24	69.08	47.66		46.0		
G 25	M36	F37		28.66		66.18	87.87	77.03		62.8		
G 26		F38		62.68		66.18	25.21	45.69		−37.2		
G 27		F39		49.93	**58.5**	66.18	69.08	67.63	**51.6**	26.2	**–34.0**	−13.37
Mean				63.45		17.88	22.1	19.98		−35.0		
LSD 5%				45.12	4.72	45.12	45.12					
* Yellow highlighting: useful positive heterosis.												
(**B**) Hybrid resistance data (bold) from the GER inheritance study and DON data (mg/kg), grouped by the father lines, original data.												
**Inbreds**	**Mother**	**G 34**	**G 30**		**G 29**	**G 28**	**G 35**	**G 33**	**G 31**	**G 32**	**G 36**	**Mean**
Father	per se	39.80	88.78		44.80	54.33	47.35	63.76	34.75	26.24	66.18	51.78
G 37	87.87	**55.50**	39.88 *		82.23 **	**89.55**	**91.04**	**78.42**	**59.77**	**114.42**	**28.66**	**71.05**
G 38	25.21	**44.33**	**81.82**		**84.24**	**57.51**	**49.01**	**60.46**	**70.31**	**76.66**	**62.68**	**65.22**
G 39	69.08	**43.24**	**37.85**		**61.17**	**105.62**	**54.82**	**70.27**	**37.91**	**25.76**	**41.25**	**53.10**
Mean	**60.72**	**47.69**	**53.19**		**75.88**	**84.22**	**64.95**	**69.72**	**55.99**	**72.28**	**44.19**	**63.12**
LSD 5% F					58.92			72.97			57.49	**15.04**
LSD 5% M												21.26
* Yellow highlighting: extra high contamination in G38 hybrids. ** Bold: hybrid data.												
(**C**) Hybrid resistance data (bold) as parental means from the GER inheritance study and DON (mg/kg), grouped by the father lines.												
**Inbreds**	**Mother**	**G34**	**G 30**		**G 29**	**G 28**	**G 35**	**G 33**	**G 31**	**G 32**	**G 36**	**Mean**
Father	per se	39.8	88.78		44.8	54.33	47.35	63.76	34.75	26.24	66.18	51.78
*G 37*	87.87	63.84 *	**88.35**		**66.33**	**71.10**	**67.61**	**75.81**	**61.31**	**57.06**	**77.03**	**69.83**
*G 38*	25.21	**32.50**	**57.02**		**35.00**	**39.77**	**36.28**	**44.48**	**29.98**	**25.73**	**45.69**	**38.50**
*G 39*	69.08	**54.44**	**78.96**		**56.94**	**61.70**	**58.22**	**66.42**	**51.91**	**47.66**	**67.63**	**60.43**
Mean	60.72	50.26	74.78		52.76	57.52	54.04	62.24	47.73	43.48	63.45	56.25
* Bold: Hybrid data.												
(**D**) ANOVA.												
**Source of Var.**		**SS**			**df**	**MS**		**F**	***p*** **-Value**		**F Crit.**	
Years		735,158.4			3	245,052.8		133.30	1.68 × 10^−45^		2.65	
Hybrids		166,584.7			44	3786.0		2.06	0.000503		1.44	
Interaction		367,728.5			132	2785.8		1.52	0.004836		1.30	
Within		330,909.7			180	1838.4						
Total		1,600,381.4			359							

**Table 4:** Combining the ability of the inbreds of maize with *F. graminearum* ear rot, with DON data (mg/kg) for one percentage of visual infection from 2017–2020. Resistance classification is based on responses of mother lines according to their artificial inoculation data.

(**A**) DON data (mg/kg) of the hybrids, with means for the eight independent data sets.												
**Hybrid**	**Mother**	**Father**	**Mother Group**	Hybrid Rate *	**Mean of Group**	**Mother Rate**	**Father Fact Rate**	**(M + F)/2**	**Mean of Group**	**Heterosis**	**Heterosis Group**	**Heterosis Change**
	Inbred		**S inbreds**	mean		Inbred		%	%	%	%	%
G 25	34	37		3.15		10.14	1.71	5.93		46.9 **		
G 26		38		0.88		10.14	2.41	6.27		**85.9**		
G 27		39		4.52		10.14	1.55	5.85		**22.6**		
G 13	30	37		1.00		1.28	1.71	1.49		**33.0**		
G 14		38		1.47		1.28	2.41	1.84		**20.0**		
G 15		39		1.47		1.28	1.55	1.41		**−3.9**		
G 4	29	37		1.90		0.83	1.71	1.27		−49.3		
		38		2.39		0.83	2.41	1.62		−47.3		
G 6		39		1.15	**1.99**	0.83	1.55	1.19	**2.99**	**3.8**	**12.4**	33.3
G 10	28	37	MS/MR inbreds	1.76		1.44	1.71	1.58		**−11.3**		
G 11		38		2.85		1.44	2.41	1.92		−48.0		
G 12		39		2.83		1.44	1.55	1.50		−89.2		
G 16	35	37		2.08		0.97	1.71	1.34		−55.3		
G 17		38		1.54		0.97	2.41	1.69		**8.9**		
G 18		39		1.16		0.97	1.55	1.26		**7.8**		
G 22	33	37		2.20		1.72	1.71	1.72		**−28.0**		
G 23		38		3.50		1.72	2.41	2.06		−69.5		
G 24		39		1.99	**2.21**	1.72	1.55	1.64	**1.63**	**−21.5**	**−34.0**	−35.3
G 19	31	37	MR inbreds	1.48		1.23	1.71	1.47		**−0.3**		
G 20		38		2.77		1.23	2.41	1.82		**−52.3**		
G 21		39		1.05		1.23	1.55	1.39		**24.7**		
G 1	32	37		1.57		0.56	1.71	1.14		**−37.8**		
G 2		38		2.18		0.56	2.41	1.48		**−46.7**		
G 3		39		3.43		0.56	1.55	1.06		**−224.7**		
G 7	36	37		0.80		1.31	1.71	1.51		**46.8**		
G 8		38		2.04		1.31	2.41	1.86		**−9.5**		
G 9		39		1.94	**1.92**	1.31	1.55	1.43	**1.46**	**−35.8**	**−37.3**	−31.1
Mean				2.04		2.16	1.89	2.03		−19.6		
LSD 5%				1.84	0.61	1.84	1.84					
* Rate: DON mg/kg/GER%, mean for eight rates. ** Yellow highlighting: useful heterosis.												
(**B**) Hybrid resistance data (bold) from the GER inheritance study, with DON data (mg/kg) for a percentage of visual infection.												
**Inbred**	**Mother**	**G 34**	**G 30**	**G 29**	**G 28**	**G 33**	**G 35**	**G 31**	**G 32**	**G 36**	**Mean**	**LSD 5%**
Father	per se	10.14	1.28	0.83	1.44	0.97	1.72	1.23	0.56	1.31	2.16	
G 37	1.71	**3.15**	**1.00**	**1.90**	**1.76**	**2.08**	**2.20**	**1.48**	**1.57**	**0.80**	**1.77**	
G 38	2.41	**0.88**	**1.47**	**2.39**	**2.85**	**1.54**	**3.50**	**2.77**	**2.18**	**2.04**	**2.18**	
G 39	1.55	**4.52**	**1.47**	**1.15**	**2.83**	**1.16**	**1.99**	**1.05**	**3.43**	**1.94**	**2.17**	
Mean	1.89	**2.85**	**1.31**	**1.81**	**2.48**	**1.59**	**2.56**	**1.77**	**2.39**	**1.59**	**2.04**	1.84
Father group				1.99			2.21			1.92		ns
(**C**) Parental means of hybrid resistance data (bold) from the GER inheritance study, with DON data as mg/kg for a percentage of visual infection.												
**Inbred**	**Mother**	**G 34**	**G 30**	**G 29**	**G 28**	**G 33**	**G 35**	**G 31**	**G 32**	**G 36**	**Mean**	
Father	per se	10.14	1.28	0.83	1.44	0.97	1.72	1.23	0.56	1.31	2.16	
G 37	1.71	**5.93**	**1.49**	**1.27**	**1.58**	**1.34**	**1.72**	**1.47**	**1.14**	**1.51**	**1.94**	
G 38	2.41	**6.27**	**1.84**	**1.62**	**1.92**	**1.69**	**2.06**	**1.82**	**1.48**	**1.86**	**2.29**	
G 39	1.55	**5.85**	**1.41**	**1.19**	**1.50**	**1.26**	**1.64**	**1.39**	**1.06**	**1.43**	**1.86**	
Mean	1.89	6.02	1.58	1.36	1.67	1.43	1.81	1.56	1.23	1.60	2.03	
Mean				**2.99**			**1.63**			**1.46**		
(**D**) ANOVA												
**Source of variance**		**SS**	**df**		**MS**		**F**	***p*** **-Value**		**F Crit.**	**LSD 5%**	
Year		319.77	3		106.59		30.12	7.94 × 10^−16^		2.65	0.39	
Genotypes		834.61	44		18.97		5.36	3.55 × 10^−16^		1.44	1.30	
Interaction		3003.09	132		22.75		6.43	1.29 × 10^−29^		1.30	2.60	
Within		637.09	180		3.54							
Total		4794.57	359									
All are significant at *p* = 0.001 or higher. SS = sum of squares, df = degree of freedom, F = F value, F crit. critical level for LSD 5%, LSD = least significant difference												

**Table 5:** Comparison of heterosis values for the different traits in the maize resistance study of GER.

Hybrid	Mother	Father	Mother Res.	Heterosis for Traits			
			group	GER%	DON (mg/kg)	DON% M/M	DON%, 8 Repl.
G19	34	37	**S inbreds**	20.45	13.06	–83.83	46.87
G20		38		10.40	−36.38	−3.43	85.90
G21		39		7.39	20.57	−90.58	22.59
G7	30	37		−2.99	54.86	−70.51	32.99
G8		38		4.04	−43.50	−311.44	19.97
G9		39		63.20	52.06	−72.76	−3.91
G4	29	37		25.24	−23.96	−119.63	−49.31
G5		38		10.99	−140.66	−77.21	−47.26
G6		39		1.69	−7.43	2.32	3.81
G1	28	37	**MS/MR inbreds**	16.05	−25.96	−47.71	−11.35
G2		38		−8.43	−44.61	−42.10	−48.04
G3		39		19.24	−71.17	−118.93	−89.25
G16	33	37		31.98	−3.43	−48.32	−27.98
G17		38		31.86	−35.91	−107.82	−69.45
G18		39		19.93	−5.80	−31.40	−21.47
G22	35	37		24.79	−34.65	−78.96	−55.30
G23		38		0.56	−35.08	−33.19	8.89
G24		39		19.62	5.84	−16.68	7.81
G10	31	37	**MR/R inbreds**	26.20	2.52	−132.42	−0.33
G11		38		−2.96	−134.53	−125.08	−52.33
G12		39		14.64	26.98	9.01	24.71
G13	32	37		−37.00	−100.53	−85.26	−37.82
G14		38		−55.43	−197.97	−80.13	−46.66
G15		39		48.30	45.96	−8.68	−224.70
G25	36	37		12.92	62.80	59.78	46.80
G26		38		−5.22	−37.16	−37.69	−9.46
G27		39		−3.31	26.18	42.38	−35.77
Mean				10.89	−24.74	−63.34	−19.63
				Traits	GER%	DON (mg/kg)	DON% Mean
				DON (mg/kg)	0.6086 **		
				DON% Mean	0.0760	0.3597	
				DONH% M/M	−0.1083	0.2063	0.0516

Green highlight: positive heterosis for all traits; orange highlight: negative heterosis for all traits; grey highlight: hybrids with positive heterosis for GER and DON. DON% M/M: rate between general means; DON%, 8 repl.: means for the eight rates. ** significant at *p* = 0.01

**Table 6:** Heterosis tests for the GER in maize, with general means for the traits tested in experimental and control hybrids, 2017–2020. Original data.

Hybrids	Traits			
	GER %	DON mg/kg	DON/% repl	DON% M/M
G1	23.60	89.55	1.76	3.79
G2	16.26	57.51	2.85	3.54
G3	18.07	105.62	2.83	5.85
G4	23.43	82.23	1.90	3.51
G5	16.72	84.24	2.39	5.04
G6	25.30	61.17	1.15	2.42
G7	17.67	39.88	1.00	2.26
G8	25.70	81.82	1.47	3.18
G9	15.05	37.85	1.47	2.52
G10	19.70	59.77	1.48	3.03
G11	14.57	70.31	2.77	4.83
G12	17.92	37.91	1.05	2.12
G13	36.78	114.42	1.57	3.11
G14	22.23	76.66	2.18	3.45
G15	11.31	25.76	3.43	2.28
G16	19.67	78.42	2.20	3.99
G17	11.15	60.46	3.50	5.42
G18	19.17	70.27	1.99	3.67
G19	14.77	55.50	3.15	3.76
G20	21.82	44.33	0.88	2.03
G21	10.58	43.24	4.52	4.09
G22	21.75	91.04	2.08	4.18
G23	16.28	49.01	1.54	3.01
G24	19.25	54.82	1.16	2.85
G25	23.92	28.66	0.80	1.20
G26	15.70	62.68	2.04	3.99
G27	23.24	49.93	1.94	2.15
GKT3275 chseck	18.05	68.69	2.41	3.81
GKT 414 chseck	17.02	61.58	1.55	3.62
P0216 chseck	14.17	38.58	1.56	2.72
Sarolta chseck	18.93	44.81	1.56	2.37
GKT 376 chseck	14.49	49.02	1.97	3.38
Csanád chseck	22.29	87.60	2.37	3.93
Mean	18.99	62.52	2.02	3.37
	GER%	DON (mg/kg)	DON/% repl.	
DON (mg/kg)	0.5433 **			
DON/%	−0.5345 ***	0.1155		
DON% Mean	−0.26825	0.6405 ***	0.6370 ***	

*** *p* = 0.001; ** p = 0.01. Hybrid names: yellow highlight, all data are lower than the column means; orange highlight, all data are above average; blue highlight, lower data than column means for GER% and DON mg/kg. Data highlight: green highlight, lower than column means; orange highlight, data are higher than column mean. DON/% repl.: means for the eight data sets counted separately; DON% M/M: rate for means across the eight data sets.

**Table 7:** Correlations between the hybrid, mother, father, and parental means for GER and heterosis values for the traits tested, 2017–2020.

GER%	Trait	Hybrid	Mother	Father	(M + F)/2
	Mother	−0.0131			
	Father	0.3051	0.0046		
	(M + F)/2	0.2652	0.4582 **	0.8909 ****	
	Heterosis	−0.5938 ***	0.3089	0.4916 ***	0.5773 ***
DON (mg/kg)	Trait	Hybrid	Mother	Father	(M + F)/2
	Mother	−0.1374			
	Father	0.0372	0.0001		
	(M+F)/2	−0.0467	0.5633 ***	0.8262 ****	
	Heterosis	−0.6572 ****	0.4038 *	0.5608 ***	0.6908 ****
DON%	Trait	Hybrid	Mother	Father	(M + F)/2
	Mother	−0.1484			
	Father	−0.3147	−0.0001		
	(M + F)/2	−0.2165	0.9734 ****	0.2289	
	Heterosis	−0.9517 ****	0.3929 *	0.3516	0.4630 **

**** *p* = 0.001; *** p = 0.01; ** p = 0.02; * p = 0.05.

**Table 8:** Temperature and precipitation data for 2017–2020 in the Kiszombor maize nursery.

**Mean Temperature °C**						
	June	July	August	September	October	Mean
2017	23.1	23.3	24.2	18.1	12.5	20.22
2018	21.6	23.6	24.6	18.9	14.3	20.6
2019	23.8	22.5	24.5	18.6	14.0	20.7
2020	21.6	22.3	23.7	19.3	12.8	19.9
**Precipitation mm**						
	June	July	August	September	October	Sum
2017	49.4	45.4	18.8	36.1	35.4	185.1.
2018	116.3	65.6	59.1	37.8	10.4	289.2
2019	111.3	47.8	23.3	30.5	27.1	240.1
2020	113.6	117.1	59.9	24.9	92.3	407.8

## Data Availability

The data that support the findings of this study are available from the corresponding author, A.M., upon reasonable request.

## Abbreviations

AER	*Aspergillus* ear rot
ANOVA	analysis of variance
DON	deoxynivalenol
DON% (M/M)	calculated between general means
DON% DON	contamination for a GER% visual infection, mean of eight epidemics (year * isolate)
FDK	*Fusarium* damaged kernel mothers
FER	*Fusarium* ear rot
FUM	fumonisin B_1_ + B_2_
_FUMB1_	fumonisin B1
G1-G4	5 maize genotypes
GCA	general combining ability
GER	*Gibberella* ear rot
GER%	*Gibberella* ear rot severity as percentage
(M+F)/2	parental mean
MR	moderately resistant
MS/MR	susceptible/moderately resistant
ns	not significant
QTL	quantitative trait locus
S	susceptible
SCA	specific combining ability
ZEA	zearalenone
